# Salicylic Acid

**Published:** 1875-07

**Authors:** 


### SELECTED ARTICLES.

### ARTICLE III.

### *Salicylic Acid.*

The dominion of "elegant pharmacy" has been extended, antiseptics and deodorisers may no longer boast of an exclusive privilege to be as disagreeable and abominable as they please; an aristocratic first cousin to carbolic acid has entered into trade, and is rapidly proving to demonstration the superiority of "blue blood." The advent, commercially, of salicylic acid as a substitute for carbolic acid may well be regarded as a great stride for those who cultivate "elegance" as well as utility and efficacy, for the former substance appears to possess a degree of antiseptic power equal, if not superior, to that of the latter; and while carbolic acid possesses a disagreeable smell and other unwelcome properties, salicylic acid appears as a crystalline powder, nearly colorless, possessing a very faint sweet taste, and almost without any injurious action on the health. We are indebted to the Germans for this conquest, whose labors have been summarized and presented before the Medical Society of the State of New York by Dr. Squibb, in the form of a "Note on Salicylic Acid," which has been printed in advance of the usual "Transactions."

Salicin is the well known vegetable principle existing in various species of the willow, poplar, and other trees and plants. Salicylic acid is a derivative of Salicin; its properties were elaborately described by Piria, and it was subsequently prepared by Lowig and Weidmann from the flowers of *Spiræa ulmaria;* and later, Procter showed that the oil of the winter green, *Gaultheria procumbens*, was salicylate of methyl, in other words, a salicylous ether. As a chemical curiosity it was studied by Gerhardt, Ettling and others.

The little that was known of the physiological and pathological effects of salicin sufficed at least to draw attention to those of its derivatives, and especially to salicylic acid, which has been the subject of occasional comment in the scientific journals for some years past. That it was peculiarly and powerfully effectual to suspend or entirely prevent fermentation and putrefaction has only quite lately been recognized by the Germans, who soon found that its natural sources, as above alluded to, were quite inadequate to enable the manufacturer to produce it in the quantities and at the price that might soon become almost a necessity. Kolbe, Professor of Chemistry at the University of Leipsic, took the matter up, and recognizing the fact that phenol or carbolic acid might be so split up as to produce, among other substances, salicylic acid, he divised a process for its manufacture which is now practically employed at a chemical works at Dresden.

Phenate of sodium is first prepared by double decomposition of phenol of soda, and well-dried carbonic anhydride is then passed through the dry powder at a temperature of 110 degrees to 250 degrees C. The carbonic anhydride combines directly with the metallic derivative of phenol, and alkaline salts of acids of a higher series are formed; among these salicylate of sodium is dissolved in water and treated with hydrochloric acid, which by double decomposi. tion sets free salicylic acid in small crystals. These crystals are washed, dissolved in hot water, and by re-crystallisation obtained in the form of a crystalline powder of a light brown color. The Germans attempt to bleach the product so obtained, and provide an article at a very high price which is sometimes quite white, but most of that in the market at a more moderate price is of a light cream color with a reddish tinge. Dr. Squibb thinks that the unbleached salicylic acid is, probably, of sufficient purity for nearly all, if not all, the practical purposes to which the acid is applied, while expensive chemical processes have to be employed in order to remove the small amount of coloring matter, which more than doubles the cost of production. Common sense seems to show that the coloring matter present is not of a kind, nor present in sufficient quantity, to interfere with the efficacy of the unbleached product, while the high price required for the more or less bleached product would shut it out from employment for most purposes, whatever might be its powers.

Dr. Squibb describes the bleached or unbleached acid as occurring in minute broken acicular crystals, which give it the appearance of a granular powder, soft and smooth under the pestle or knife, but somewhat rough or resinous when rubbed between the fingers. This powder is odorless and nearly tasteless. It has, however, a sweetish and astringent after taste, with slight acridity in the fauces, but none in the mouth ; and though tasteless it leaves a disposition or inclination to expectorate which continues for some time.

Salicylic acid is very difficultly soluble in cold water, but easily dissolved by hot water, alcohol and ether. An aqueous solution containing from 0.2 to 0.4 per cent. of salicylic acid may be obtained by cooling a hot solution, when the excess crystallises out. The acid is far more soluble in water, containing a small portion of neutral salt. In Germany a solution is used for surgical purposes which contains one gramme of the acid dissolved in fifty grammes of water containing three grammes of sodium phosphate. Salicylic acid is decomposed into phenol and carbonic anhydride.

Its compounds with the bases or salts seem difficult to make, but salicylate of zinc, a crystalline salt moderately soluble in water, and salicylate of quinine, amorphous, insoluble in water but soluble in alcohol, have already been prepared in Germany.

Dr. Squibb very properly points out that it is, in all probability, a purely accidental, although a very curious circumstance that a substance of long and well established character as an anti-ferment should offer a molecular constitution so well adapted to be broken up into a still more powerful anti-ferment, for there is no relation whatever, either in composition, or chemical or physical properties between carbolic acid and salicylic acid, except in their effects by similar or altogether different reactions. Accordingly it must not be hastily assumed that in salicylic acid we have simply carbolic acid under a new name, but the compound must be experimentally tested, compared, and then judged on its merits. Numerous experiments reveal the fact that salicylic acid is a powerful antiseptic; indeed it is asserted to be far more powerful and effective in smaller quantities than any other antiseptic. Consequently its innocuous character, and the absence of odour and taste which characterise it, make it immeasurably superior to carbolic acid, which possesses qualities sufficient to restrict its application within very narrow limits. Other advantages which salicylic acid is said to possess beyond all other antiseptics are, first, that it may be used in quantities sufficient to be completely effectual for surgical purposes, and yet devoid of any irritating action on the living tissues, nor does it produce inflammation, nor any caustic or corrosive effect in any quantity. Although the very small quantities that are effectual are quite neutral, it is admitted that large quantities may be irritant or painful, but not beyond what may be described as a stimulant. Secondly, it is said to have power over processes of decomposition which are beyond the reach of all other antiseptics or anti-ferment since it entirely suspends the chemical vitality which causes the production of the volatile oils in mustard, and bitter almonds, the effect of diastase, etc. Thirdly, it has no poisonous effect in any reasonable quantity.

Brewer's yeast does not effect a solution of glucose to which one thousandth part of salicylic acid has been added. Mustard flour, which, when treated with a little tepid water, almost immediately develops a sharp odor of essence of mustard, remains quite inodorous if a small quantity of salicylic acid be added. The action of emulsia, the ferment contained in sweet or bitter almonds or amygdalin contained in bitter almonds only, whereby essence of bitter almonds is produced, is entirely prevented by salicylic acid. Fresh milk mixed with 0.04 per cent. of salicylic acid and allowed to stand at a temperature of 80° F. in an open vessel took thirty-six hours longer to curdle than the same quantity similarly exposed in a pure state. The neutral salts of salicylic acid do not, according to Kolbe, produce this effect, but only the free acid. Beer containing one-thousandth part of salicylic acid did not become sour when exposed to the air, neither did it exhibit any trace of that cryptogamic vegetation which appears on the surface of spoiled beer. Eggs which had been plunged in a solution of salicylic acid for one hour remained unaffected for three months. Fresh meat on which the acid had been sprinkled remained sweet for several weeks. It prevents or arrests the souring of worts, washes and beers of the brewers, and the putrefactive changes which are so troublesome to the glue manufacturers. Urine to which some salicylic acid had been added was on the third day, still clear, and without ammoniacal odor. According to the results obtained by Professor Neugebouer fermentation may be prevented by adding 100 grammes of salicylic acid to 1,000 litres of beer. The same author recommends the use of a very weak solution of salicylic acid to rinse out the wine casks, and thus hinder the formation of mould. Small quantities of salicylic acid would also, in the estimation of Professor Neugebouer, if added to wine, prevent that after fermentation which is the principle cause of muddiness in wines, and perhaps check all the wine diseases produced by the growth of fungi. Professor Kolbe finds that a half a gramme of salicylic acid is sufficient to check the further progress of fermentation produced by the action of 5 grammes of beer yeast on a solution of 120 grammes of sugar in 1 litre of water. It has been suggested that such facts as these will indicate the quantities of salicylic acid to be used in the manufacture of fruit essences, champagnes, beer for exportation; and by way, perhaps of reassurance to those who might object to be dosed continously with a chemical of which we know so little as of salicylic acid, it is stated that Professor Kolbe could take without disturbing his digestion or general health from 1 to 1.25 grammes of salicylic acid per diem either in water or spirit. Surely, however, an isolated experiment of this kind is not enough to establish the harmlessness of the substance so as to warrant the recommenda tion of the substance for general employment in the preparation of articles of food.

Moreover Professor Kolbe proposes to use this substance for the prevention of putrefaction in water stored on board of ships the object to be attained either by dissolving the salicylic acid in the water itself in the maximum proportion of 1 to 20,000, or by covering the bung-holes of the water-casks with cotton impregnated with salicylic acid. Would the salicylic acid be quite harmless if used in the former way? A suggestion which we should feel much less hesitation in adopting personally is that a capital dentrifice may be made by perfuming an alcoholic solution of salicylic acid with oil of wintergreen. Used in small quantities, mixed with luke-warm water, acts as an effectual preserver of the teeth; or an excellent tooth powder may be prepared with salicylic acid. A "sprinkling-powder" for the feet has also been proposed, which acts without checking the perspiration. It should be composed of salicylic acid, talc-powdered soap, and starch. Besides removing odor, it communicates an agreeable softness to the feet.

The phosphate of sodium, solution of salicylic acid was employed by Professor Thiersch to promote the growth of skin over granulated surfaces. Or salicylic acid used alone or mixed with starch was used upon contused or incised wounds, and in operations, with excellent general results, destroying the fetid odor of cancerous surfaces and pyæmic ulcerations. Again, Dr, Fehling, of the Leipsic Lying-in

Hospital, reports its use instead of carbolic acid for disinfecting the hands, sponging per vaginum, for sprinkling puerperal ulcers, etc. For these purposes a solution is employed of from 1 in 300 to 1 in 900, or a mixtnre with starch-flour in the proportion of 1 to 5.

An emulsion, apparently for internal use, is recommended by Professor Wunderlich, of

| | Parts. |
|---|---|
| Salicylic Acid, - - - - - - | 1 |
| Sweet Almond Oil, - - - - - | 20 |
| Gum Arabic, - - - - - - | 10 |
| Syrup of Almonds, - - - . | 25 |
| Orange Flower Water - - - - - | 45 |

We cannot over-estimate the importance of that branch of experimental inquiry which deals with such questions as the influence of agents like carbolic and salicylic acids on septic and zymotic poisoning. These investigations should be pushed to their farthest limit, even if not one in ten put forward by chemistry repay the labor of investigation, for it is certainly in this direction of research that medicine must look with greatest hope of success to control those abnormal vital processes which so far may be modified but not stopped. The phenols will always retain their importance among this class of agents, surpassing as they do all that have been tried before them. If salicylic acid should prove another step in advance, the gain will be great, more especially as indicating discoveries which may enable us to wield an undreamed of power against the most frightful and hitherto unconquerable ills of humanity.—*The Chemist and Druggist.*

